# Immunological profile of lactylation-related genes in Crohn’s disease: a comprehensive analysis based on bulk and single-cell RNA sequencing data

**DOI:** 10.1186/s12967-024-05092-z

**Published:** 2024-03-23

**Authors:** Jingtong Wu, Yinyin Lv, Pei Hao, Ziyi Zhang, Yongtian Zheng, Ermei Chen, Yanyun Fan

**Affiliations:** 1 Department of Gastroenterology, Zhongshan Hospital of Xiamen University, School of Medicine, Xiamen University, Xiamen, 361004 Fujian People’s Republic of China; 2 Institute for Microbial Ecology, School of Medicine, Xiamen University, Xiamen, 361004 Fujian People’s Republic of China; 3 Department of Digestive Disease, School of Medicine, Xiamen University, Xiamen, 361004 Fujian People’s Republic of China

**Keywords:** Lactylation, Crohn’s disease, Immune infiltration

## Abstract

**Background:**

Crohn's disease (CD) is a disease characterized by intestinal immune dysfunction, often accompanied by metabolic abnormalities. Disturbances in lactate metabolism have been found in the intestine of patients with CD, but studies on the role of lactate and related Lactylation in the pathogenesis of CD are still unknown.

**Methods:**

We identified the core genes associated with Lactylation by downloading and merging three CD-related datasets (GSE16879, GSE75214, and GSE112366) from the GEO database, and analyzed the functions associated with the hub genes and the correlation between their expression levels and immune infiltration through comprehensive analysis. We explored the Lactylation levels of different immune cells using single-cell data and further analyzed the differences in Lactylation levels between inflammatory and non-inflammatory sites.

**Results:**

We identified six Lactylation-related hub genes that are highly associated with CD. Further analysis revealed that these six hub genes were highly correlated with the level of immune cell infiltration. To further clarify the effect of Lactylation on immune cells, we analyzed single-cell sequencing data of immune cells from inflammatory and non-inflammatory sites in CD patients and found that there were significant differences in the levels of Lactylation between different types of immune cells, and that the levels of Lactylation were significantly higher in immune cells from inflammatory sites.

**Conclusions:**

These results suggest that Lactylation-related genes and their functions are closely associated with changes in inflammatory cells in CD patients.

**Supplementary Information:**

The online version contains supplementary material available at 10.1186/s12967-024-05092-z.

## Introduction

Inflammatory bowel disease (IBD) is an idiopathic inflammatory disease of the intestinal tract, including ulcerative colitis (UC) and Crohn's disease (CD), whose incidence and prevalence are increasing worldwide, and which affects people of all ages in all aspects of life [1]. The etiology of CD is unclear and it is now generally accepted that it is a chronic inflammatory bowel disease due to a multifactorial immune dysfunction of the intestinal mucosa mediated by genetics, environment, intestinal mucosal barrier dysfunction and intestinal microcosmology [2]. IBD patients present with clinical symptoms such as diarrhea and mucopurulent blood stools, which in severe cases can lead to hospitalization and surgery, and although the mortality rate is low, lifelong treatment is required [3]. The incidence of IBD is increasing worldwide and there is an urgent need for more effective treatments.

Immune abnormalities are thought to be one of the most important factors contributing to CD, and it has now been suggested that perturbations in the metabolic pathways of immune cells may have a greater impact on the progression of CD [4]. Meanwhile, dysregulation of organic acid metabolism in CD patients has long been of concern, and a significant increase in intestinal lactate levels has been reported in CD patients [5].

Lactic acid was long thought to be a waste product of glycolysis [6], while in 2019, Prof. Zhao's team reported for the first time that Lactylation of lactate-derived histone lysine residues is an epigenetic modification that directly stimulates gene transcription in chromatin [7]. Subsequently, in 2022, Prof. Ye made progress in analyzing protein lactylation using tandem mass spectrometry and found that protein lactylation is widespread and may severely affect non-histone protein functions [8]. Recently, in 2023, Prof. Yuan found that meiotic recombinant protein 11 (MRE11), which undergoes lactylation modification, plays an important role in the process of chemotherapeutic resistance [9]. Contemporary, in vitro experiments have confirmed that high levels of lactic acid interfere with T-cell migration and induce T-cells to produce more pro-inflammatory cytokines such as IL-17, perpetuating local chronic inflammation [10]. In rheumatoid arthritis, high levels of lactate in the inflamed synovium are translocated via MCT1 to CD4 T cells and reprogram glycolysis via the STAT17/RORγt pathway, inducing IL-17 expression and exacerbating tissue inflammation [11]. In contrast to its role in significantly inducing and promoting inflammation in rheumatoid arthritis (RA), lactate plays a protective role in mouse models of immune hepatitis and pancreatitis [12]. While CNS macrophages are highly glycolytic in multiple sclerosis (MS), blocking macrophage lactate secretion in an experimental autoimmune encephalomyelitis (EAE) model of MS reduced leukocyte infiltration and clinical severity of EAE [13]. In addition, animal studies have shown that lactic acid consumption via gut bacteria can alleviate intestinal inflammation in a mouse model of DSS colitis [14], but topical treatment with lactic acid prevents TNBS-induced colitis [15]. Therefore, elevated intestinal lactate may be related to the progression of CD, but its specific function needs to be further elucidated. Meanwhile, the expression of lactate-related genes and their related functions in CD have not been reported.

In this study, we applied an integrated analysis based on bulk RNA sequencing (RNA-seq) and single-cell RNA sequencing (scRNA-seq) data to identify the hub genes for lactylation in the gut of CD patients. The relationship between the CD immune microenvironment (IME) and lactylation levels was also elucidated. It is expected to open a new promising approach for identifying potential biomarkers for CD diagnosis and to provide guidance for future pathogenesis studies.

## Methods

### Identification of differences in gut expression genes between IBD and healthy individuals

Three CD-related gene expression matrices (GSE16879, GSE75214 and GSE112366) were obtained from the GEO database (https://www.ncbi.nlm.nih.gov/geo/) and data from a total of 226 CD patients and 43 healthy individuals were used for the subsequent analyses (18 CD patients and 6 healthy individuals from GSE16879, 67 CD patients and 11 healthy individuals from GSE75214, 141 CD patients and 26 healthy individuals from GSE112366). Batch effects between different datasets were removed using the R package ‘sva’, and datasets were normalized using the R package ‘preprocessCore’. Overall differences in gene expression between CD patients and healthy individuals were compared using the “limma” package and visualized using the “ggplots2” and “heatmap” packages.

### Identification of lactylation-related hub genes in CD patients

34 Lactylation-related genes obtained from gene ontology (http://geneontology.org//) and previously reported (PMID: 36,092,712), of which 16 genes with significant expression differences between CD patients and healthy individuals were included in further analyses. We used the STRING database to predict Protein–Protein Interaction Networks (PPIs) of the 16 obtained genes. The least absolute shrinkage and selection operator (LASSO) is a type of multivariate linear regression in which the coefficients are continuously shrunk by adding a penalty function to streamline the model to avoid covariance and overfitting, and is commonly used in candidate gene screening [16, 17]. LASSO regression was implemented using the “glmNETs” R package to filter down to 11 genes. Random Forest classifier and Support Vector Machine (SVM) analyses were implemented using the “randomForest” and “kernlab” R packages, and the top 10 genes were retained. Then, the Lactylation-related hub genes were identified by taking the intersection of the results of LASSO regression, Random Forest, and SVM analyses. The area under the receiver operator characteristic (ROC) curve (AUC) was calculated to evaluate the diagnostic value of the hub gene in CD using the R package “pROC”. GO and Kyoto Encyclopedia of the Genome (KEGG) pathway analyses were performed using the R package “clusterProfile”.

### Relationship between lactylation hub genes and immunological traits

We used the ssGSEA algorithm through the R package “GSVA” to comprehensively assess the immune cell infiltration of each sample in the study. We then analyzed the correlation between hub genes and infiltrating immune cells using Spearman analysis.

### Gene set enrichment analysis

We analyzed the co-expression of hub genes using Spearman analysis. Gene set enrichment analysis was performed on the Hallmark gene sets (http://software.broadinstitute.org/gsea/msigdb/) using the R package “clusterProfiler” based on the significant co-expression gene of hub genes (P < 0.05). Finally, we used the regnetwork database to predict miRNAs and transcription factors upstream of genes and constructed networks using Cytoscape software.

### scRNA-seq analysis

We obtained scRNA-seq data from the GEO database (GSE134809) containing 11 CD patients (paired ileal resection samples from inflamed and non-inflamed areas) and processed the data using the R package “Seurat”. We excluded cells with a percentage of mitochondrial genes higher than 15%, cells with a percentage of ribosomal gene expression higher than 3%, cells with a percentage of erythrocyte gene expression higher than 0.1%, and cells expressing less than 200 genes and more than 7,500 genes to maintain high quality data. We eliminated batch effects between samples using the R package 'harmony' and normalized the data using the ScaleData function, followed by Principal Component Analysis (PCA) on the scaled data. We used the RunUMAP function for umap. We used the FindAllMarkers function to find differential genes in each cluster. Finally, we used the “SingleR” package to annotate cell types.

We used the “GSVA” package to evaluate the lactylation enrichment scores of each cell in the scRNA-seq dataset and score each cell based on the HALLMARK pathway, KEGG_MEDICUS subset of Canonical pathways and PID subset of CP Canonical pathways. Then, we analyzed the correlation between the pathway scores and the Lactylation scores.

### Animal experience

Eight-week-old C57BL/6 WT male mice were purchased from the Xiamen University Laboratory Animal Centre and housed in a dedicated pathogen-free facility at 25 °C with a 12/12 h light/dark cycle, with blinded sampling in a randomised design (7 mice per group). For the mouse model of colitis, 3% DSS (molecular weight 36–50 kDa; MP Biomedicals) was added to the drinking water for 5 days, followed by 3 days of normal drinking water, and control mice received normal drinking water throughout the experiment. At the end of the experiment, mice were sacrificed and colon tissues collected. RNA was extracted using Trizol reagent (15,596,026, Invitrogen) and complementary DNA was synthesized using the Hifair^®^ II 1st Strand cDNA Synthesis Kit (11121ES60, Yeasen) according to the manufacturer’s instructions. Real-time PCR analysis was performed using Hieff® qPCR SYBR Green Master Mix (11203ES03, Yeasen) and primer sequences are shown in Additional file 1: Table S1.

## Results

### The overall expression profile of intestinal in CD patients

**Fig. 1:**
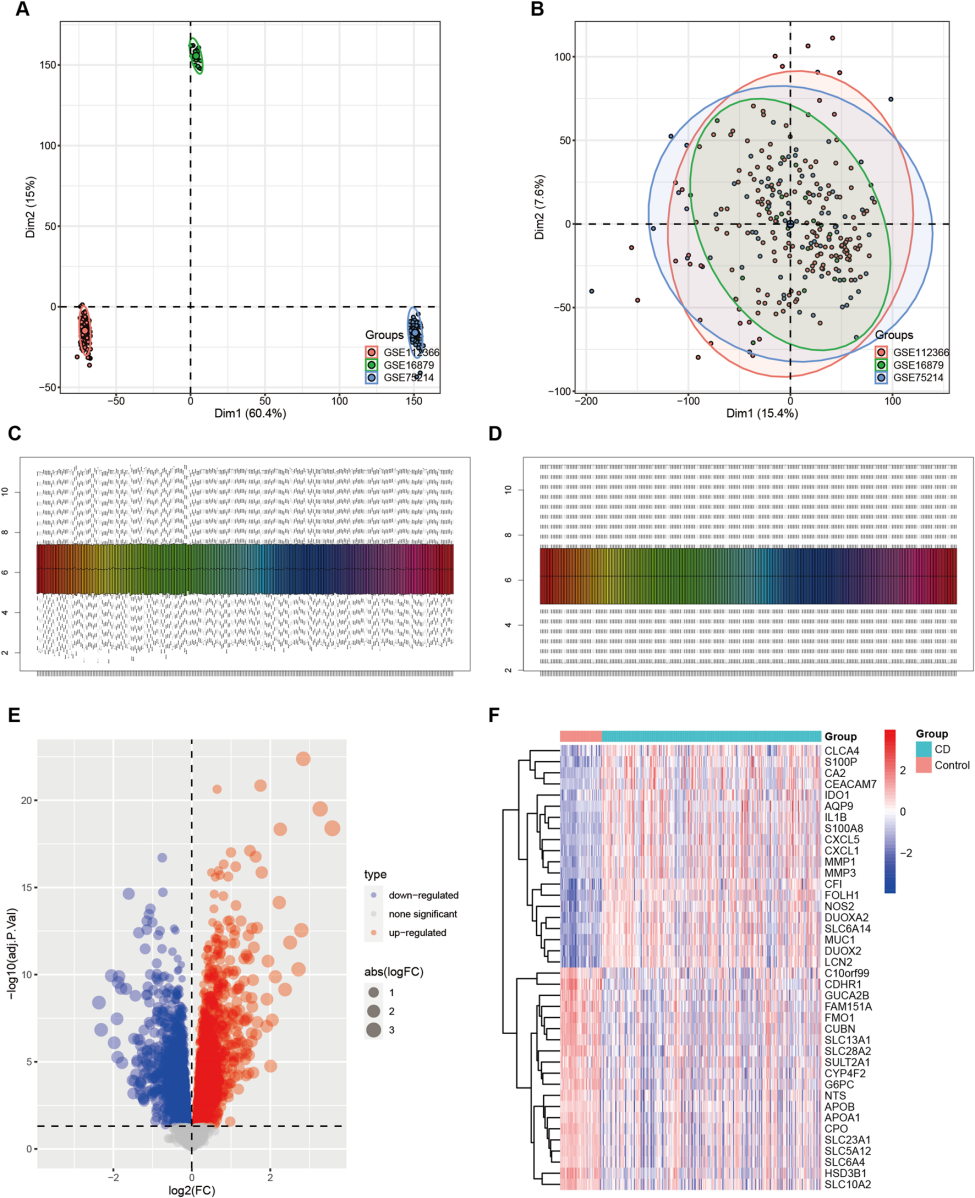
Combined post-correction analysis of three GEO matrices. PCA plots showing clustering of samples based on gene expression profiles, raw data batch-corrected **A** before and **B** after batch-corrected; total DEGs **C** before and **D** after normalisation; **E** analysis of variance volcano plots, where grey represents genes with adj. P. Val > 0.05; **F** heat map showing genes that differed between CD and control (top 20)

After obtaining three CD-related datasets (GSE16879, GSE75214, and GSE112366) and removing any batch effects, we acquired expression profiles for 226 CD patients and 43 healthy controls (Fig. 1A–D). Analysis of variance revealed 2751 up-regulated genes and 3064 down-regulated genes in the gut of CD patients (Fig. 1E, F). These differentially expressed genes were further subjected to functional and GO analysis, which highlighted that “positive regulation of cell adhesion”, “apical part of cell”, and “phospholipid binding” were strongly associated with CD (Additional file 2: Fig. S1A–C). Additionally, KEGG analysis showed strong correlation of CD with "Focal adhesion" and "PI3K-Akt signaling" (Additional file 2: Fig. S1D).

### The expression feature of Lactylation-related genes

**Fig. 2:**
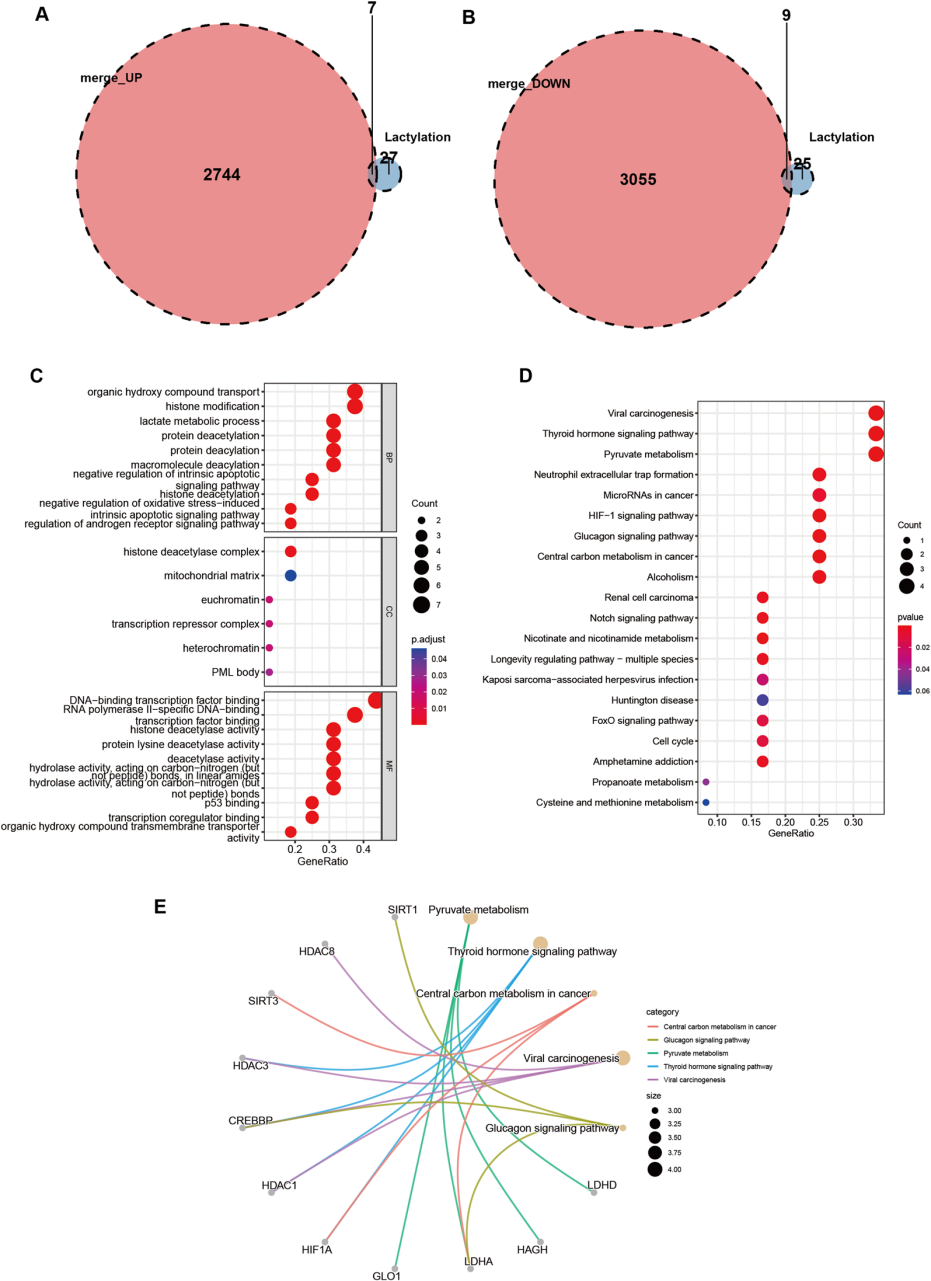
Differential expression of Lactylation genes. Wayne diagram showing the intersection of **A** up-regulated genes and **B** down-regulated genes with Lactylation genes; use of the intersected genes for **C** GO functional clustering analysis and **D** KEGG functional clustering analysis; **E** correlation between the intersected genes and KEGG results (Top 5)

After obtaining the aforementioned profiles, we investigated the 34 genes associated with lactylation and their respective expression variances. Our findings reveal that seven genes associated with lactylation were noticeably up-regulated in patients with CD, in contrast to nine down-regulated genes (Fig. 2A, B, Additional file 3: Fig. S2). To further validate the expression profiles of these 16 genes in colitis, we established a DSS-induced mouse model and verified by qPCR using mouse colon tissue that the expression profiles of these 16 genes in the mouse model of colitis were consistent with the results of the bioinformatics analysis (Additional file 4: Fig. S3). We conducted functional analyses on 16 genes displaying differential expression in relation to Lactylation. Our GO analysis demonstrated significant alterations in “histone deacetylation”, “histone modification”, and “histone deacetylase complex” (Fig. 2C). In addition, our KEGG analysis revealed significant alterations in “Pyruvate metabolism”, “Neutrophil extracellular trap formation”, and the “HIF-1 signaling pathway” (Fig. 2D). Finally, we utilized the STRING database to forecast protein interaction networks for the 16 lactation-related genes displaying differential expression (Fig. 2E).

### Identification of hub genes of lactylation

**Fig. 3:**
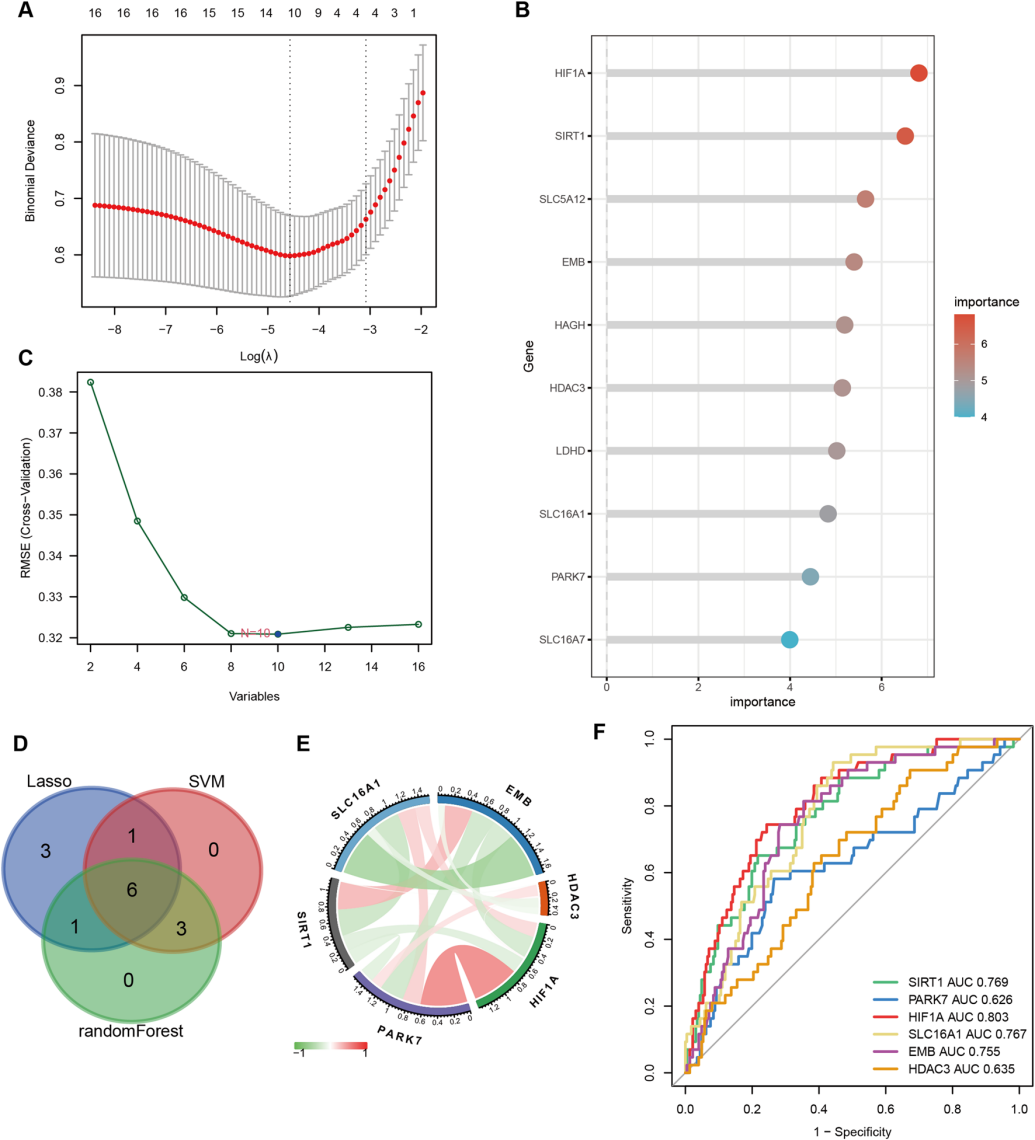
Identification of six hub genes of lactylation. **A** LASSO regression screening to obtain 11 genes; **B** random forest to select 10 genes according to their importance; **C** SVM support vector machine to screen 10 genes; **D** intersection of the results obtained by the above three methods to obtain six hub genes; **E** correlation between the six hub genes, where red colour represents a positive correlation and green colour represents a negative correlation; **F** ROC curves of six genes for predicting the occurrence of the disease

To identify the hub genes of Lactylation-related genes objectively, we conducted lasso regression on 16 differentially expressed genes and uncovered 11 genes (Fig. 3A). We utilized Random Forest Analysis and the SVM-RFE algorithm to rank Lactylation-related genes and identified the top 10 genes (Fig. 3B, C). By taking the overlap of the genes obtained from these three methods, we identified six genes as the hub genes of Lactylation, namely EMB, HDAC3, HIF1A, PARK7, SIRT1, and SLC16A1 (Fig. 3D, E). Their potential in distinguishing between CD patients and healthy individuals was assessed using ROC, and HIF1A, SIRT1, SLC16A1, and EMB were found to exhibit reasonable discriminatory capabilities (Fig. 3F).

### Functional analysis of hub genes in CD patients

**Fig. 4:**
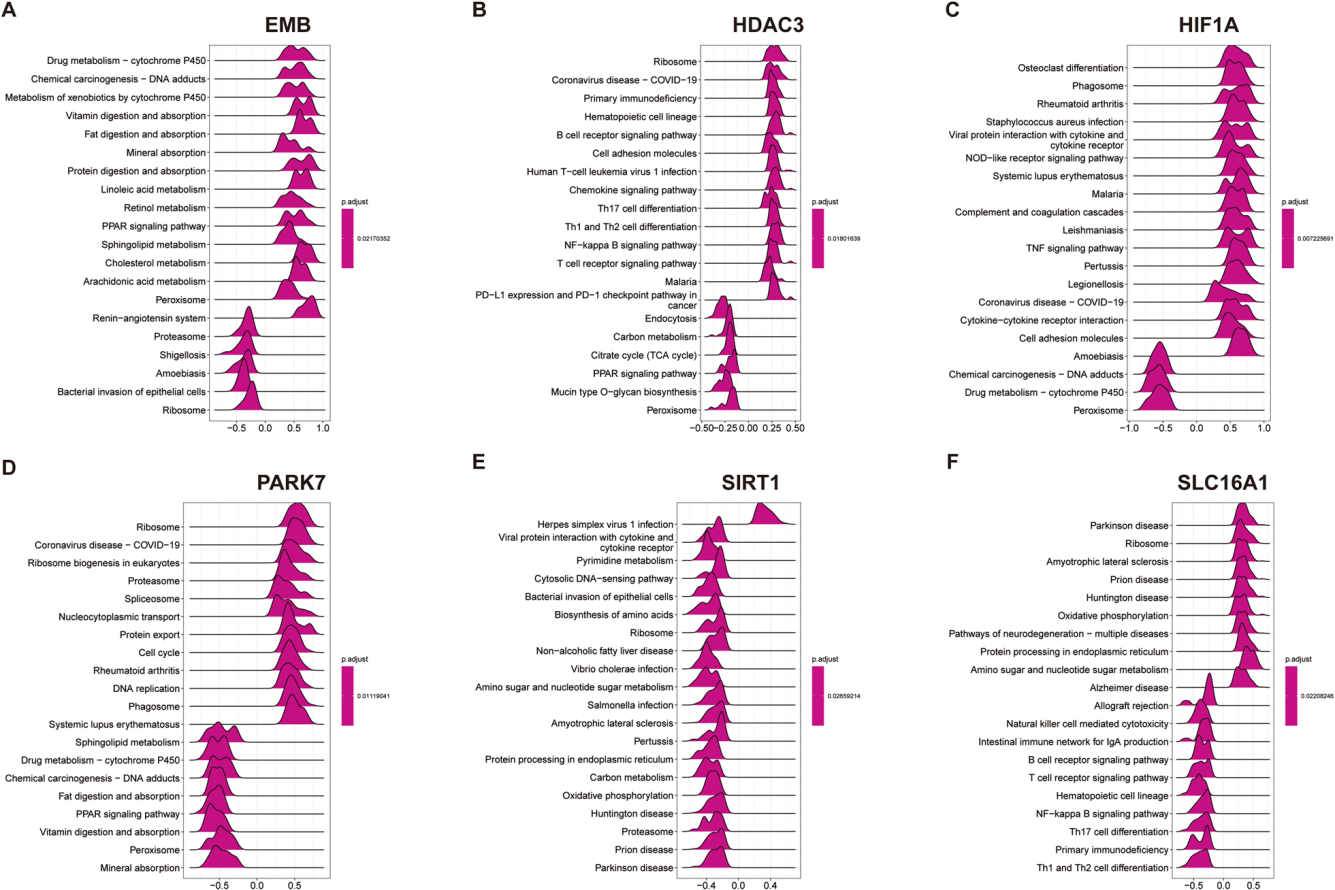
The correlation between hub gene expression and KEGG pathway. The GSEA analysis of KEGG (top20) based on the correlation analysis result of **A** EMB, **B** HDAC3, **C** HIF1A, **D** PARK7, **E** SIRT1 and **F** SLC16A1

After identifying the hub genes, we conducted a correlation analysis between each of these six hub genes and all other genes, followed by a GSEA analysis based on the correlation results (Additional file 5: Fig. S4). EMB expression showed positive correlation with the absorption of various nutrients and negative correlation with bacterial invasion of the intestinal epithelium (Fig. 4A). HDAC3 expression showed a positive correlation with the B cell receptor signaling pathway, Th cell differentiation, and NF-kappa B signaling pathway, whilst displaying a negative correlation with the TCA cycle (Fig. 4B). HIF1A expression showed a positive correlation with the NOD-like receptor signaling pathway and TNF signaling pathway (Fig. 4C). PARK7 expression showed a negative correlation with the digestion and absorption of fat and vitamins, whilst displaying a positive correlation with the Proteasome and Cell cycle (Fig. 4D). SIRT1 expression showed negative correlation with Proteasome, Oxidative phosphorylation, and Salmonella infection (Fig. 4E). On the other hand, SLC16A1 expression exhibited negative correlation with Natural killer cell-mediated cytotoxicity, B cell receptor signaling pathway, and T cell receptor signaling pathway, while exhibiting positive correlation with Oxidative phosphorylation and Ribosome (Fig. 4F).

### The expression of hub genes correlated with immunological characteristics in CD patients

**Fig. 5:**
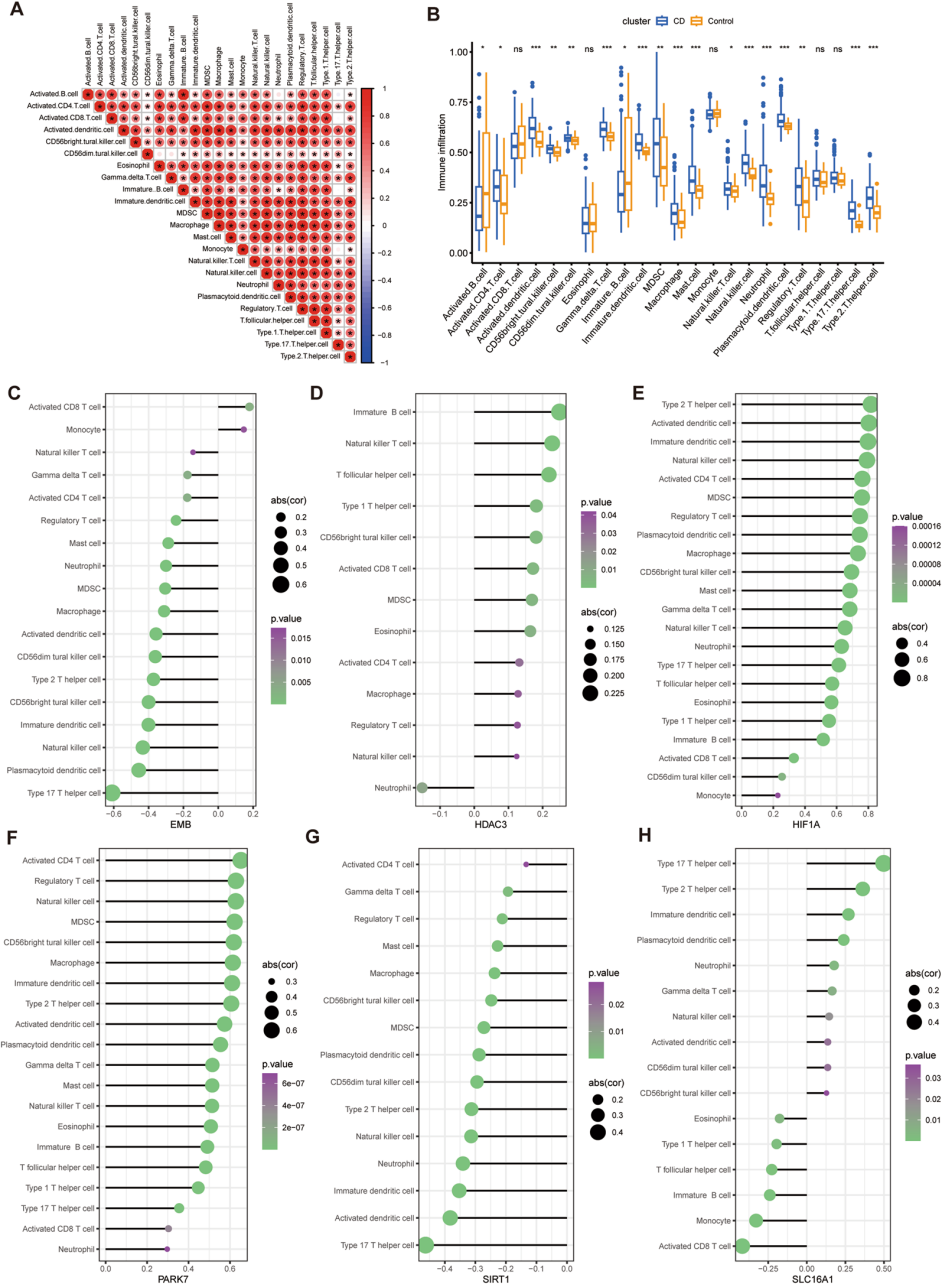
Correlation of hub genes with immune cell infiltration. **A** Correlation between immune cell infiltration; **B** difference in immune cell infiltration between CD and healthy controls; demonstration of correlation between immune cell infiltration and **C** EMB, **D** HDAC3, **E** HIF1A, **F** PARK7, **G** SIRT1 and **H** SLC16A1

We conducted an analysis of the immune cell infiltration levels in both CD patients and healthy controls. Our findings indicated a significant increase in the level of infiltration of 16 out of 23 immune cells in the intestines of CD patients, whereas there was a significant decrease in the levels of 2 out of 23 immune cells (Fig. 5A, B). We identified a significant negative correlation between EMB expression and the level of infiltration of 16 of 23 immune cells, additionally, there was a positive correlation between the number of activated CD8 T cells and monocytes (Fig. 5C). The expression of HDAC3 demonstrated a notable positive correlation with the infiltration level of 12 out of 23 immune cells and a negative correlation with the number of neutrophils (Fig. 5D). Correspondingly, the expression of HIF1A showed significant positive correlation with the degree of infiltration of 22 of the 23 immune cells (Fig. 5E), while the expression of PARK7 was significantly positively correlated with the level of infiltration of 20 of the 23 immune cells (Fig. 5F). SIRT1 expression exhibited a significant negative correlation with the infiltration levels of 15 out of 23 immune cells (Fig. 5G), while SLC16A1 expression displayed a significant positive correlation with the infiltration levels of 12 out of 23 immune cells and a negative correlation with 6 out of 23 immune cells (Fig. 5H).

### Identification of 11 cell clusters in CD patients using scRNA-seq data

**Fig. 6:**
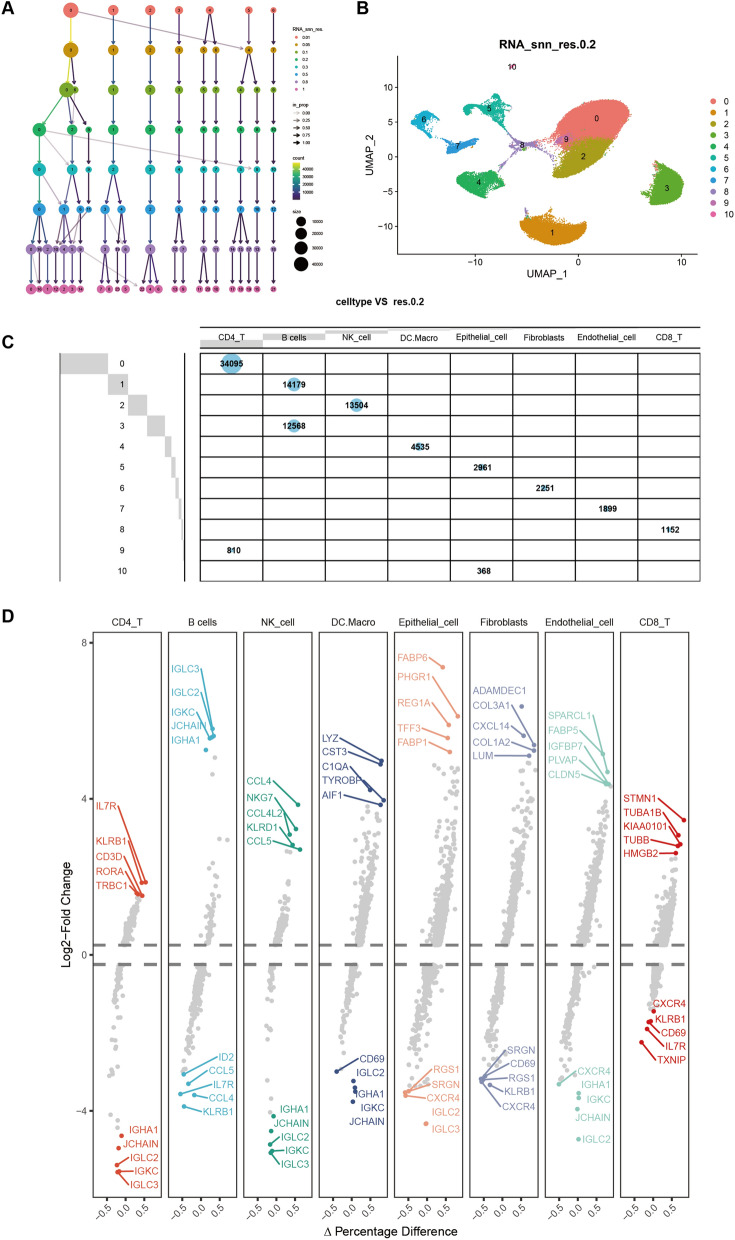
Single cell RNA sequence analysis. **A** Cluster tree diagram of cell grouping at different resolutions; **B** cells were grouped into 11 separate clusters by UMAP; **C** number and assignment of cells in each cluster; **D** differentially expressed genes per cluster (top5)

As demonstrated in Fig. 1, 88,322 cells were analyzed following quality control criteria and standardization of CD scRNA-seq data (Additional file 6: Fig. S5A–E). A total of 20,527 genes were examined, and the leading 25 genes are displayed in Additional file 6: Fig. S5F. Next, Uniform Manifold Approximation and Projection (UMAP) was utilized to reduce dimensions, successfully sorting cells into 11 distinct clusters (res = 0.2) (Fig. 6A, B). Following this, the “SingleR” functionality was used for cell annotation, leading to the annotation and representation of eight cell types: CD4 T cell, B cells, NK cell, DC/Macrophage, epithelial cell, Fibroblasts, endothelial cell, and CD8 T cells (Fig. 6C). Differential expression analysis was conducted, uncovering a complete count of 6659 marker genes from all 11 clusters, as illustrated in Fig. 6D.

### Single-cell analysis validated the Lactylation interacts with the immune in CD

**Fig. 7:**
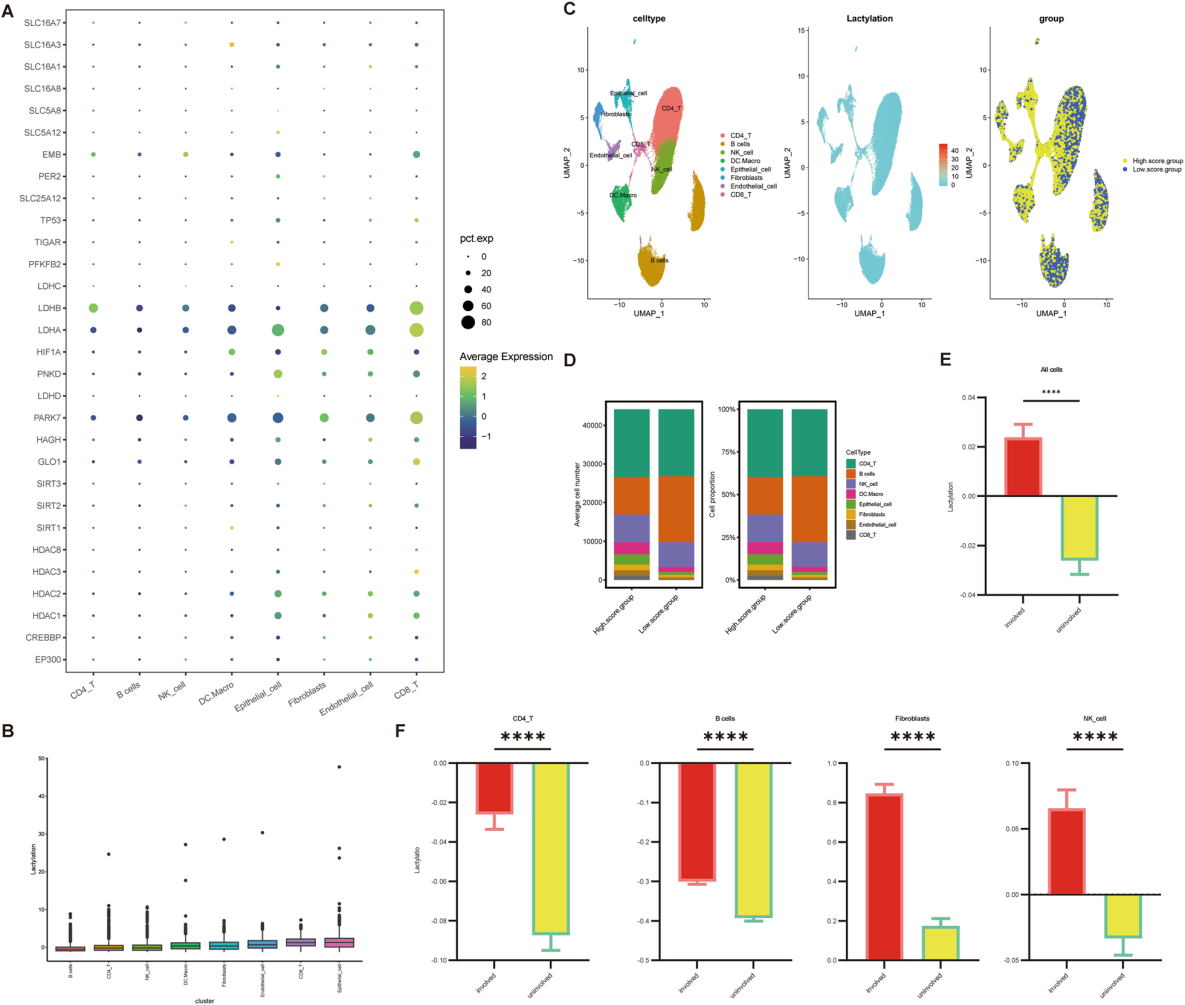
Lactylation profiling of CD patients based on single cell sequencing. **A** Expression of genes within the lactylation gene set across cells; **B** scoring of each cell based on the lactylation gene set using the R package GSVA, scoring the level of the gene set across cell types; **C** lactylation gene set scoring as shown by UMAP (high and low level groups based on median); **D** number and percentage of cells of each cell type between the high and low expression groups of lactylation gene set scoring; **E** overall difference in lactylation gene scores between inflammatory and non-inflammatory sites in CD patients and **F** cell types difference

We assessed the Lactylation level of individual cells by examining the expression of 34 lactylation-related genes in various cell types (Fig. 7A). According to the figure, epithelial cells, CD8 T cells, and endothelial cells had relatively high Lactylation level scores (Fig. 7B, C). However, B cells, CD4 T cells, and NK cells displayed relatively low scores. The cells were subsequently sorted by Lactylation level and separated into high and low Lactylation groups based on the median value. We discovered that B cells exhibited comparatively lower Lactylation levels, whereas DC/Macrophage, epithelial cells, Fibroblasts, endothelial cells, and CD8 T cells showed relatively higher Lactylation levels (Fig. 7D). Finally, the cells were categorized and analyzed by regions of inflammation, revealing that Lactylation levels were generally greater in inflamed areas than in non-inflamed ones (Fig. 7E). Additionally, Lactylation levels of specific cell types, such as CD4 T cells, B cells, epithelial cells, fibroblasts, endothelial cells, and NK cells, were significantly higher in inflammatory regions (Fig. 7F).

### Functional clustering analysis identifies key pathways associated with Lactylation

**Fig. 8:**
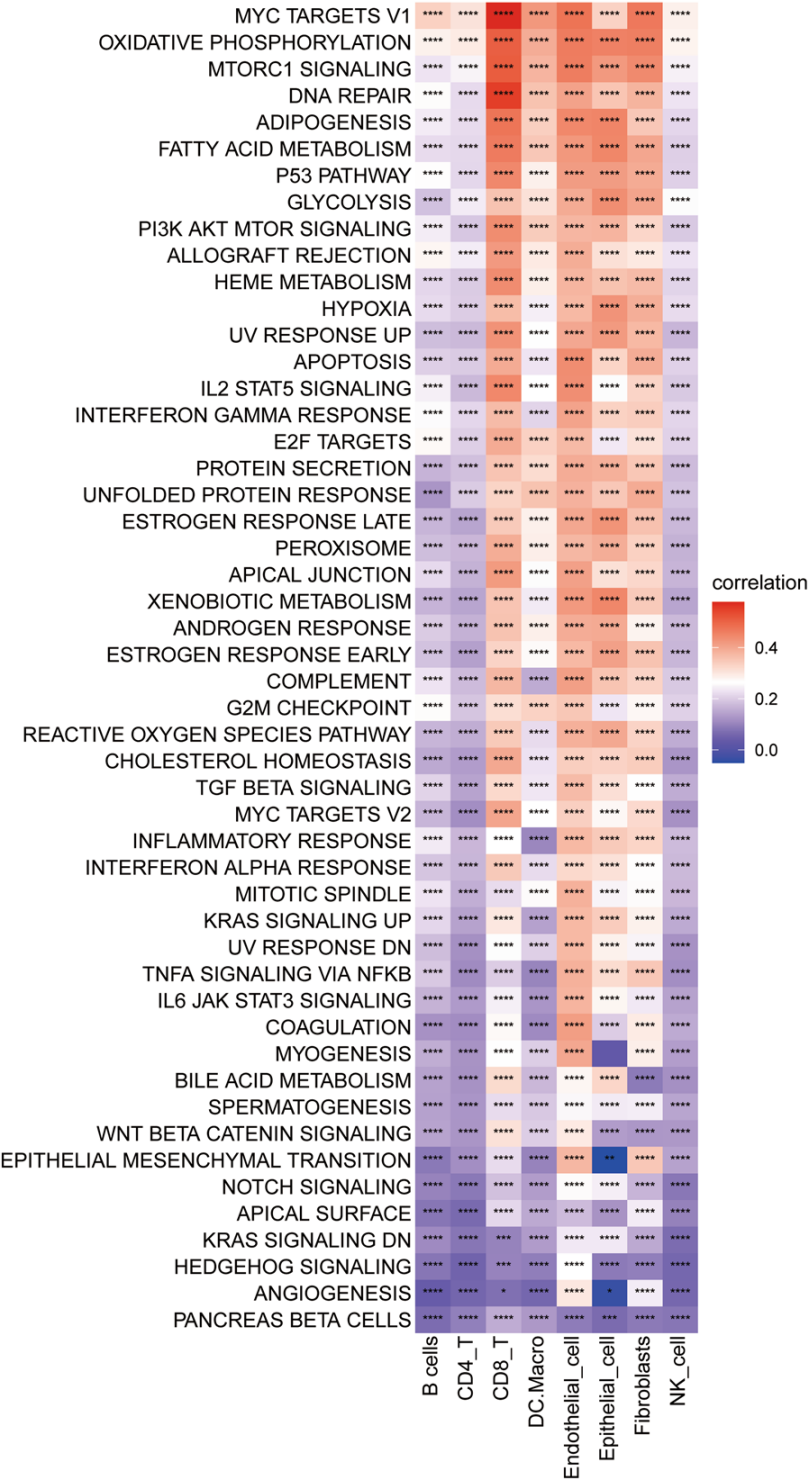
Heatmap of correlation between lactylation score and the HALLMARK pathway in each cell type. Each cell was scored based on the HALLMARK pathway (MsigDB database) and the correlation of the Lactylation score with the HALLMARK pathway score is shown

To further identify the key pathways associated with lactylation, each cell was scored based on the dataset from MSigDB using the R package GSVA, and the correlation between the HALLMARK pathway scores and the lactylation level was analyzed in different cell types, and it was found that the “OXIDATIVE PHOSPHORYLATION”, “MYC_TARGETS_V1” and “MTORC1_SIGNALING” pathways showed the strongest correlation in all cells (Fig. 8). Based on the KEGG dataset, we found that “KEGG HUNTINGTONS DISEASE” and “KEGG PYRUVATE METABOLISM” showed the strongest correlation with the lactylation level, and we also found that “KEGG TIGHT JUNCTION” and “KEGG APOPTOSIS” also showed strong correlation with the lactylation level (Additional file 7: Fig. S6). Based on the PID dataset, we found that “PID HDAC CLASSI PATHWAY”, “PID HDAC CLASSIII PATHWAY” and “PID MYC ACTIV PATHWAY” had the strongest correlation with the lactylation level (Additional file 8**:** Fig. S7).

## Discussion

CD encompasses a complex set of disease phenotypes that are primarily characterized by abnormal activation of inflammation in the intestinal mucosa [18]. It is currently believed that the pathogenesis of CD is based on the activation of intestinal inflammation by a combination of factors such as the environment, a western diet or disturbances in the intestinal flora, based on a susceptible genetic background [19]. There are many studies on the effects of diet and gut flora metabolites on gut immunity [20–22], but there are few studies on the effects of the body’s own metabolite, lactate, and its subsequent lactation-associated changes on gut immunity. In this study, we used RNA sequencing data to identify six hub genes for Lactylation and analyzed the relationship between these hub genes and the level of immune cell infiltration. We also carried out a comprehensive analysis of the scRNA-seq data and found that the level of Lactylation in cells is closely related to aberrant activation of immune system function.

Currently, the diagnosis of Crohn's disease requires a systematic evaluation of endoscopic, histological, radiological and clinical data [23], and early diagnosis of CD significantly reduces the risk of adverse clinical outcomes [24]. By analyzing RNA sequencing data, we found that HIF1A, SIRT1, SLC16A1 and EMB in the hub genes have the potential to be used as markers to distinguish CD patients from healthy individuals. HIF1A, hypoxia-inducible factor, is a major regulator of glycolytic metabolism and cytoplasmic lactate levels [25]. Recent studies have found that lactate inhibits CNS autoimmunity by stabilizing HIF1A in dendritic cells promoting NDUFA1L1 expression thereby limiting mitochondrial reactive oxygen species production. Our findings that HIF1A expression correlates with the NOD-like receptor signaling pathway, the TNF signaling pathway and almost all immune cell infiltrations suggest that HIF1A has a strong correlation with a pro-inflammatory phenotype in intestinal immunity and that further experimental studies are needed to clarify its specific function and mechanism. The SIRT1 (sirtuin 1) protein is a highly conserved NAD-dependent deacetylase that links transcriptional regulation to intracellular energy and is involved in the orchestration of cellular functions [26]. The gene is currently thought to have some inhibitory inflammatory function and is downregulated in inflammatory states [27]. Our analyses revealed that SIRT1 expression levels were decreased in the intestines of CD patients and showed a negative correlation with Oxidative phosphorylation and a negative correlation with the infiltration of pro-inflammatory cells, such as Th17, which is consistent with previous reports. SLC16A1 encodes a monocarboxylate transporter protein and catalyzes the proton-linked transport of monocarboxylates (e.g. L-lactate, pyruvate and ketone bodies) across the plasma membrane [28]. In the current report, SLC16A1-mediated lactate efflux is critical for maintaining Treg cell function and inhibiting plasma cell-like dendritic cell (pDC) activation [29, 30]. Our analyses also revealed that SLC16A1 expression was negatively correlated with natural killer cell-mediated cytotoxicity, B cell receptor signaling pathway and T cell receptor signaling pathway, which is consistent with existing literature, but we also found that SLC16A1 expression was positively correlated with Th17 cell infiltration, suggesting that SLC16A1-related mechanisms need to be further explored. EMB (mbigin), a member of the immunoglobulin superfamily, promotes the localization of monocarboxylate transporter proteins to the plasma membrane [31–33]. We found that EMB expression was decreased in the intestine of CD patients and negatively correlated with the level of bacterial invasion of the intestinal epithelium and the infiltration of pro-inflammatory immune cells such as Th17 cells, suggesting that EMB may play a role in maintaining intestinal immune homeostasis and is a protective factor for CD.

Metabolomic data confirm that immune abnormalities in CD patients are highly correlated with gut metabolic disturbances [20, 34, 35], and recent epigenetic studies suggest that lactate-induced changes in histone lactylation have a significant impact on cellular reprogramming and mediate immune cell modification [36, 37]. Therefore, using single-cell sequencing data, we performed a comprehensive analysis of cellular lactylation levels at inflammatory and non-inflammatory sites in CD patients and found that lactylation levels at inflammatory sites were significantly higher than those at non-inflammatory sites in CD patients, which may be related to high lactate aggregation at inflammatory sites. Among immune cells, B cells, CD4 T cells and NK cells had relatively low overall lactylation levels, but there was a significant difference between the lactylation levels of cells at inflammatory and non-inflammatory sites. Thus, the modulation of lactylation on intestinal B cells, CD4 T cells and NK cells in CD patients needs to be further investigated. Finally, we analyzed the correlation between lactate levels and functional pathways in different cell types. HELLMARK-based analysis revealed that lactylation levels were highly correlated with oxidative phosphorylation in all cells, which is consistent with previous reports based on intestinal epithelial cells [38]. The results of the KEGG analysis showed a correlation between pyruvate metabolism and lactylation levels, which are known to have a strong association with intestinal homeostasis [39]. In addition, the results showed that lactylation levels correlated with both tight junction and apoptotic pathways, which have long been shown to correlate with IBD pathogenesis [40]. Results from the PID dataset also detected MYC correlations consistent with HELLMARK pathway, suggesting that lactonization levels may be related to MYC function.

This study has limitations. Although our study shows that lactylation-associated genes may serve as diagnostic markers for CD and that increased levels of lactylation immune cells at sites of inflammation may be involved in the pathogenesis of CD, further animal and clinical studies are needed to validate our findings.

In conclusion, we performed a comprehensive analysis to explore the impact of lactylation-related genes on CD and identified four hub genes that could serve as novel diagnostic markers for CD patients. At the same time, we described the lactylation of intestinal immune cells in CD patients based on single-cell data, providing new insights into the relationship between lactylation and CD that may be useful for future research.

### Supplementary Information

**Additional file 1: Table S1.** Primer used for RT-PCR.

**Additional file 2: Figure S1.** GO annotation and KEGG enrichment analysis of differential genes. GO functional enrichment analysis of differential genes, **A** show the pathways annotated in Biological Process (BP), **B** Cellular Component (CC), and **C** Molecular Function (MF); (D) KEGG pathway enrichment analysis results.

**Additional file 3: Figure S2.** Differential expression of 16 lactation-related differential genes in CD and control. **A** Volcano plot, **B** Heat map and **C** Box plot showing the expression of 16 differential genes in CD and control, respectively.

**Additional file 4: Figure S3.** Quantitative PCR validation of 16 lactation-related differential genes in the colitis mouse model. Quantitative RT-PCR analysis of 16 lactation-related differential genes in colon tissues of the colitis mouse model and its blank control group (n = 7 per group). Data are presented as mean ± SEM. Data are presented as mean ± SEM. *p < 0.05, **p < 0.01, ***p < 0.001, ****p < 0.0001.

**Additional file 5: Figure S4.** Correlation analyses of the six genes with all genes were performed. Heatmaps were used to show the expression of positively correlated top50 genes, respectively.

**Additional file 6: Figure S5.** Quality control of CD single-cell RNA sequencing data. nFeature_RNA and nCount_RNA of single-cell sequencing data **A** before and **B** after exclusion; the percentage of expression of mitochondrial genes, ribosomal genes and erythrocyte genes of single-cell sequencing data **C** before and **D** after exclusion; **E** correlation between nFeature_RNA and nCount_RNA; **F** display of genes with higher percentage of single-cell sequencing (top 25).

**Additional file 7: Figure S6.** Heatmap of correlation between lactylation score and the KEGG pathways in each cell type. Each cell was scored based on the KEGG_MEDICUS subset of Canonical pathways (MsigDB database) and the correlation of the Lactylation score with the KEGG pathway score is shown.

**Additional file 8: Figure S7.** Heatmap of correlation between lactylation score and the PID pathways in each cell type. Each cell was scored based on the PID subset of Canonical pathways (MsigDB database) and the correlation of the Lactylation score with the PID pathway score is shown.

## Data Availability

The datasets used and/or analyzed during the current study are available from the corresponding authors upon reasonable request.
