# Supplementary material for: Immunological profile of lactylation-related genes in Crohn’s disease: a comprehensive analysis based on bulk and single-cell RNA sequencing data
Source: J Transl Med. 2024 Mar 23;22:300. doi: 10.1186/s12967-024-05092-z (PMC10960451; doi:10.1186/s12967-024-05092-z)
Supplement: Supplementary file 1 — Additional file 1: Table S1. Primer used for RT-PCR. [file 12967_2024_5092_MOESM1_ESM.docx]

| Table S1. Primer used for RT-PCR | |
| --- | --- |
| Primer | Sequence |
| mu-HDAC3-F | AACCTCATCGCCTGGCATTGAC |
| mu-HDAC3-R | GTAGTCCTCAGAATGGAAGCGG |
| mu-SIRT3-F | GCTACATGCACGGTCTGTCGAA |
| mu-SIRT3-R | CAATGTCGGGTTTCACAACGCC |
| mu-CREBBP-F | CACCATCTGTGGCTACTCCTCA |
| mu-CREBBP-R | GGTTTCAGCACTGGTCACAGAG |
| mu-SIRT1-F | GGAGCAGATTAGTAAGCGGCTTG |
| mu-SIRT1-R | GTTACTGCCACAGGAACTAGAGG |
| mu-GLO1-F | CCCTGCTATGAAGTTCTCGCTC |
| mu-GLO1-R | GAGTCTCGTCATCTTCAGTGCC |
| mu-HAGH1-F | GGTTTATGGAGGTGATGACCGC |
| mu-HAGH1-R | CAGATGTGTCCCGAAGTATGGC |
| mu-LDHA-F | ACGCAGACAAGGAGCAGTGGAA |
| mu-LDHA-R | ATGCTCTCAGCCAAGTCTGCCA |
| mu-EMB-F | GGAGAAAAGGAACTAAGAGGGAC |
| mu-EMB-R | CACACTTCAGCACAGTAGAATCC |
| mu-SLC5A12-F | TCCACACCTCTCCGACAAACTG |
| mu-SLC5A12-R | ATGAGGGATGCAACCACAGCCA |
| mu-HDAC8-F | GTCAGCCAAGAAGGTGATGAGG |
| mu-HDAC8-R | ACACTTCCCGTCAATCAGGCAC |
| mu-LDHD-F | GGACTTTGTGGAGGCTCTGAAG |
| mu-LDHD-R | CCAAACCACAGCATCAGGAGGT |
| mu-SLC16A1-F | GACCATTGTGGAATGCTGCCCT |
| mu-SLC16A1-R | CGATGATGAGGATCACGCCACA |
| mu-SLC16A7-F | CTACCAGGTTCTCCAGTGCTGT |
| mu-SLC16A7-R | ACGACTGTTCCGCTGGCTATGT |
| mu-HDAC1-F | TGAAGCCTCACCGAATCCGCAT |
| mu-HDAC1-R | TGGTCATCTCCTCAGCATTGGC |
| mu-HIFA1-F | CCTGCACTGAATCAAGAGGTTGC |
| mu-HIFA1-R | CCATCAGAAGGACTTGCTGGCT |
| mu-PARK7-F | ACGATGTGGTGGTTCTTCCAGG |
| mu-PARK7-R | CTGCACAGATGGCAGCTATGAG |
| mu-GAPDH-F | CATCACTGCCACCCAGAAGACTG |
| mu-GAPDH-R | ATGCCAGTGAGCTTCCCGTTCAG |
