# Supplementary figures and images for: Immunological profile of lactylation-related genes in Crohn’s disease: a comprehensive analysis based on bulk and single-cell RNA sequencing data
Source: J Transl Med. 2024 Mar 23;22:300. doi: 10.1186/s12967-024-05092-z (PMC10960451; doi:10.1186/s12967-024-05092-z)

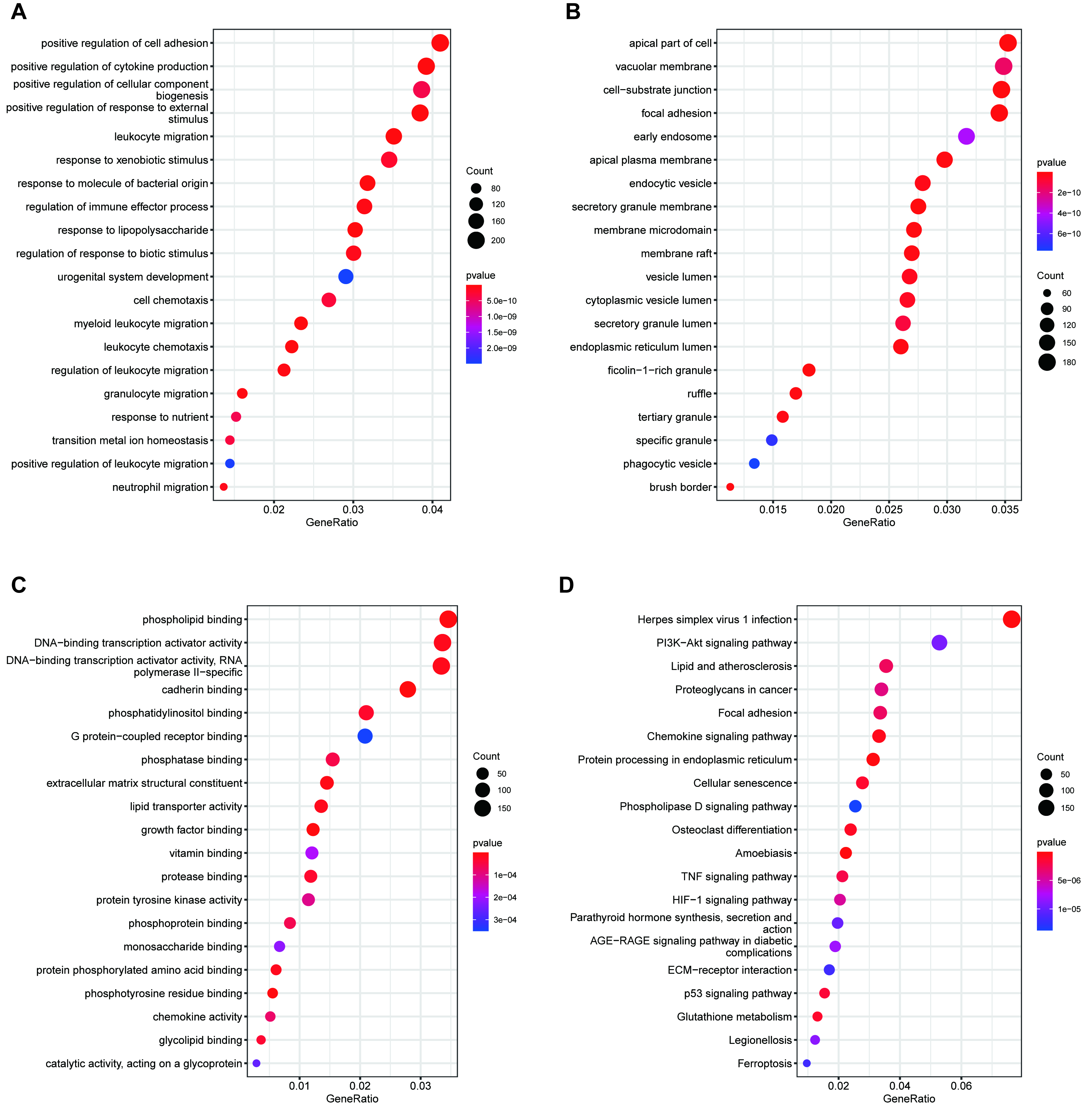

Supplement: Supplementary file 2 — Additional file 2: Figure S1. GO annotation and KEGG enrichment analysis of differential genes. GO functional enrichment analysis of differential genes, A show the pathways annotated in Biological Process (BP), B Cellular Component (CC), and C Molecular Function (MF); (D) KEGG pathway enrichment analysis results. [file 12967_2024_5092_MOESM2_ESM.tif]

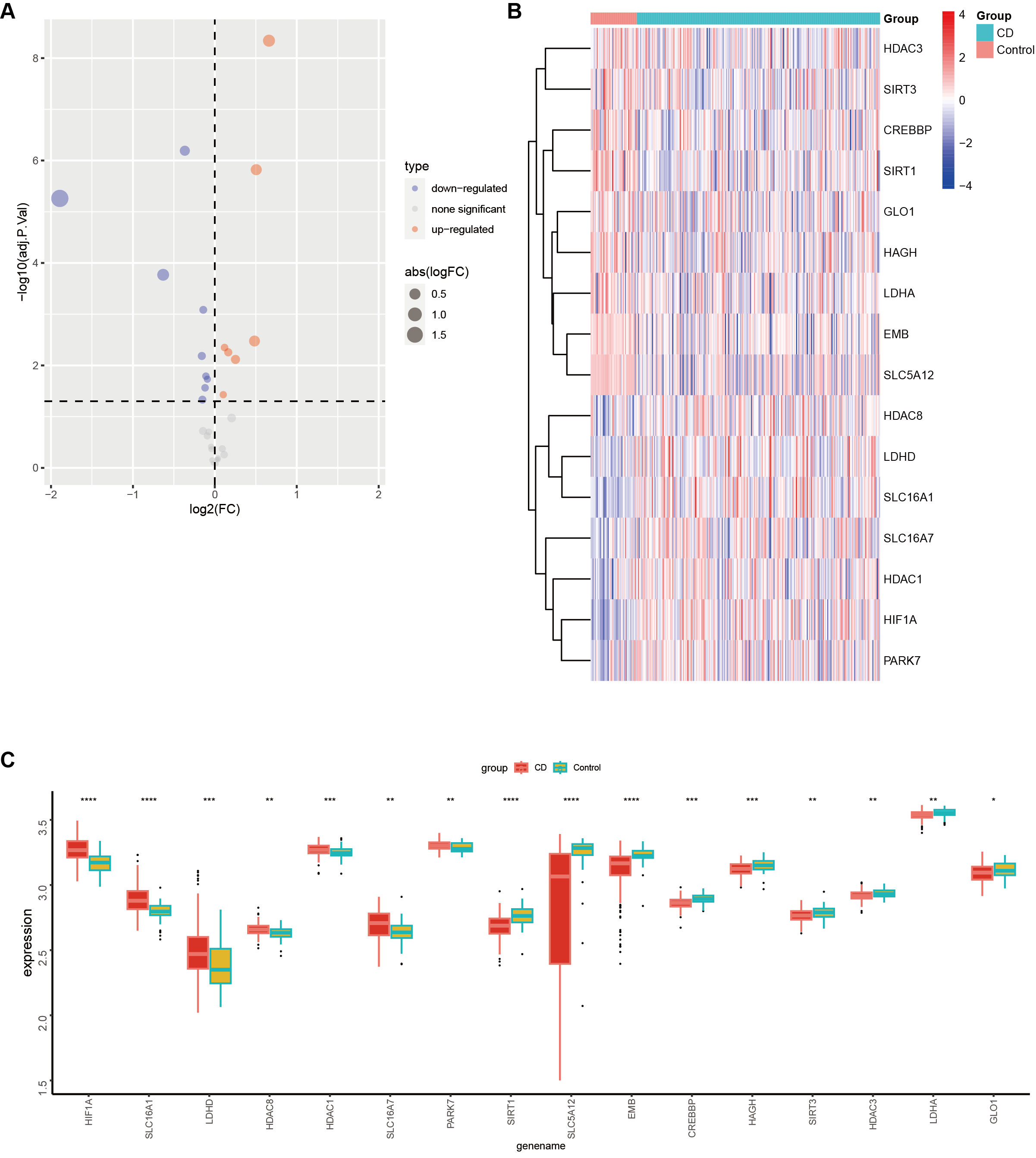

Supplement: Supplementary file 3 — Additional file 3: Figure S2. Differential expression of 16 lactation-related differential genes in CD and control. A Volcano plot, B Heat map and C Box plot showing the expression of 16 differential genes in CD and control, respectively. [file 12967_2024_5092_MOESM3_ESM.tif]

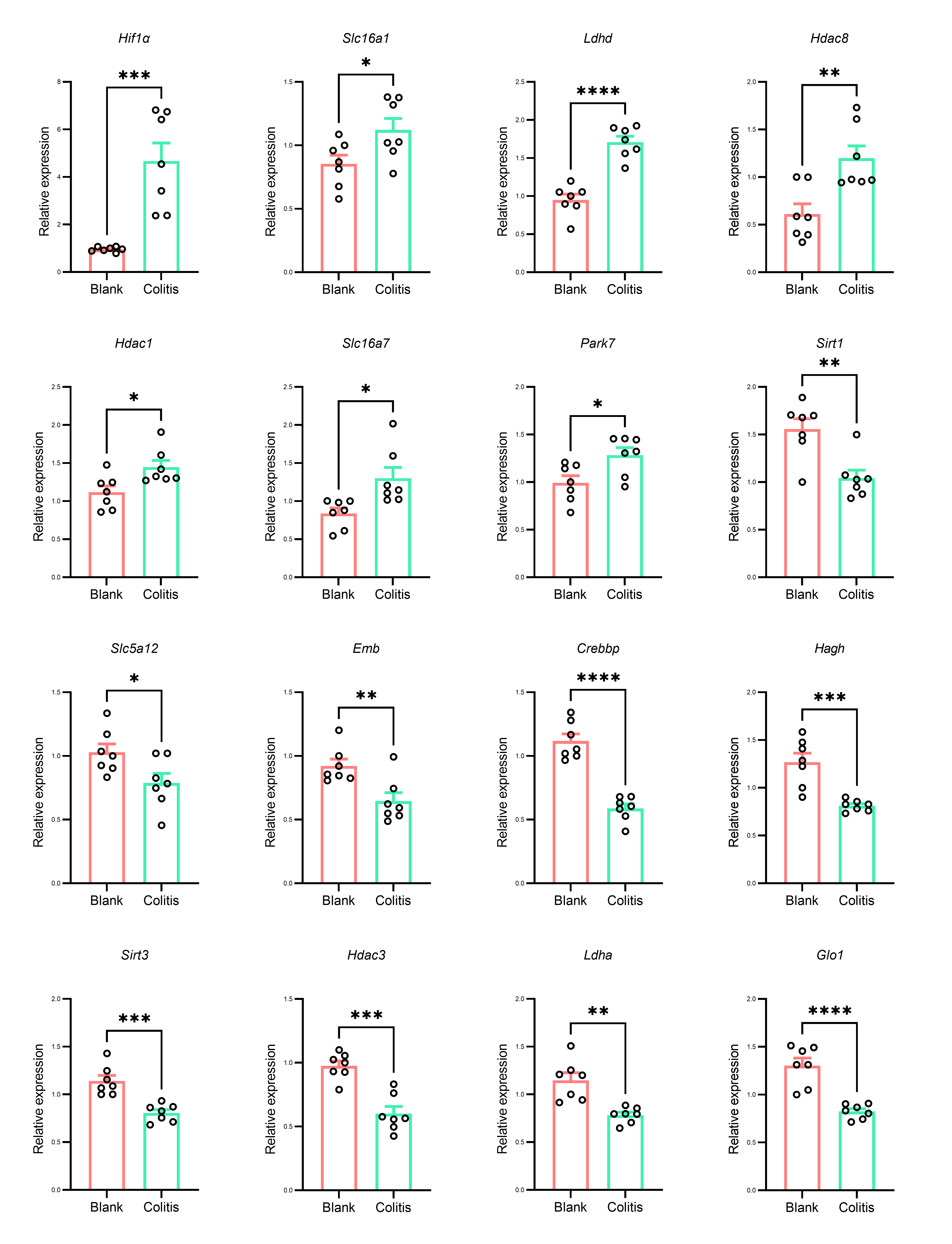

Supplement: Supplementary file 4 — Additional file 4: Figure S3. Quantitative PCR validation of 16 lactation-related differential genes in the colitis mouse model. Quantitative RT-PCR analysis of 16 lactation-related differential genes in colon tissues of the colitis mouse model and its blank control group (n = 7 per group). Data are presented as mean ± SEM. Data are presented as mean ± SEM. *p < 0.05, **p < 0.01, ***p < 0.001, ****p < 0.0001. [file 12967_2024_5092_MOESM4_ESM.tif]

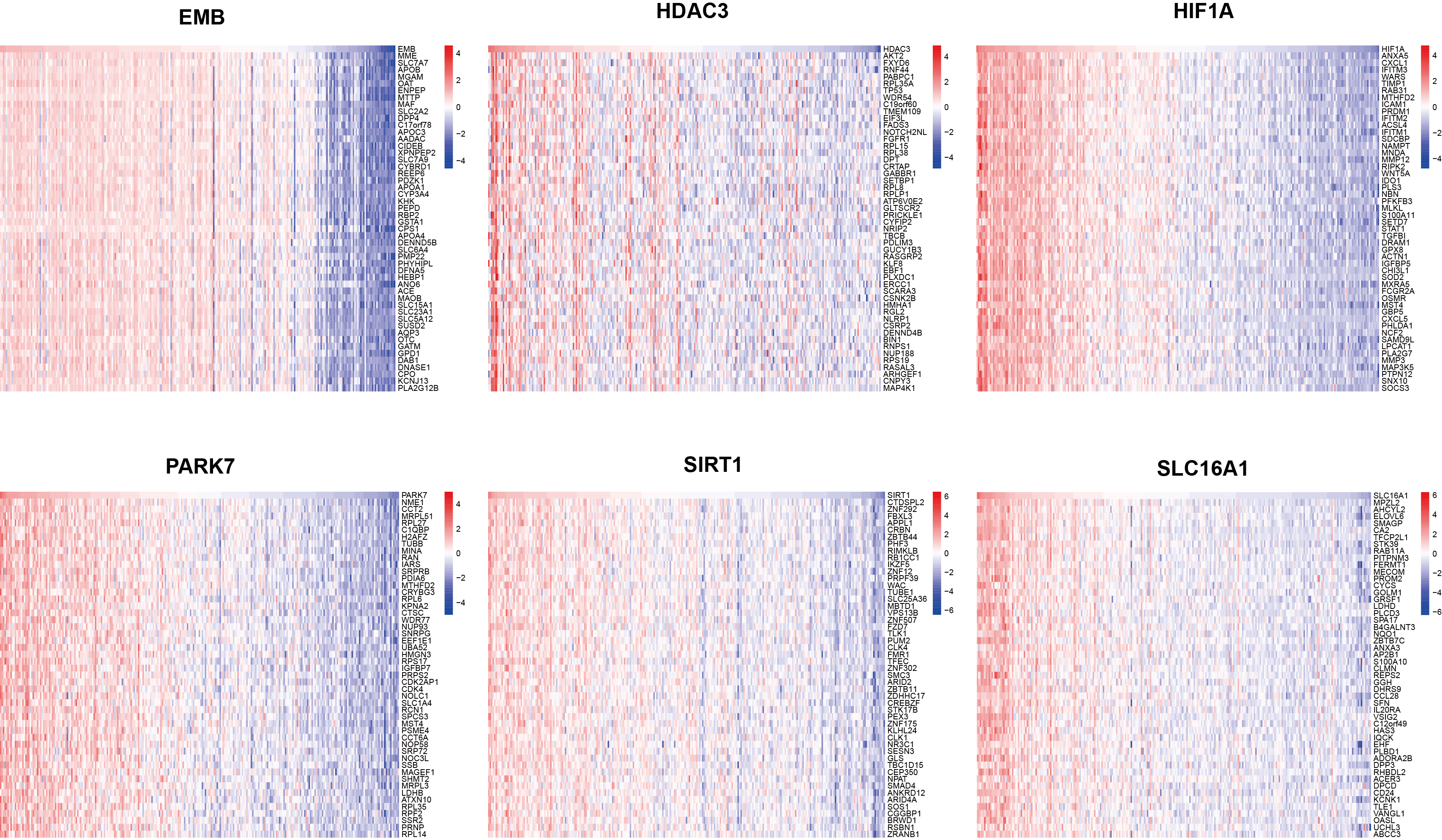

Supplement: Supplementary file 5 — Additional file 5: Figure S4. Correlation analyses of the six genes with all genes were performed. Heatmaps were used to show the expression of positively correlated top50 genes, respectively. [file 12967_2024_5092_MOESM5_ESM.tif]

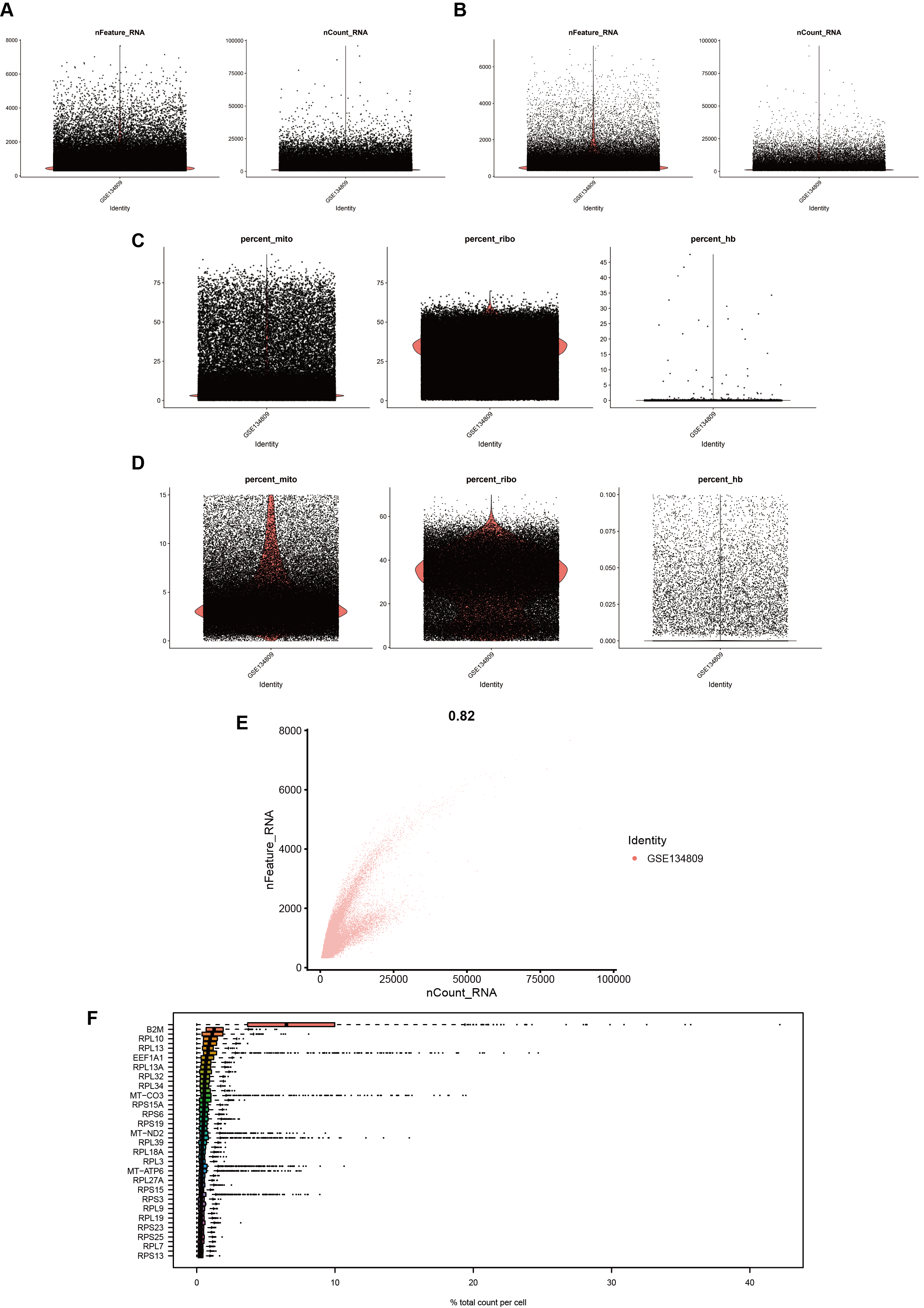

Supplement: Supplementary file 6 — Additional file 6: Figure S5. Quality control of CD single-cell RNA sequencing data. nFeature_RNA and nCount_RNA of single-cell sequencing data A before and B after exclusion; the percentage of expression of mitochondrial genes, ribosomal genes and erythrocyte genes of single-cell sequencing data C before and D after exclusion; E correlation between nFeature_RNA and nCount_RNA; F display of genes with higher percentage of single-cell sequencing (top 25). [file 12967_2024_5092_MOESM6_ESM.tif]

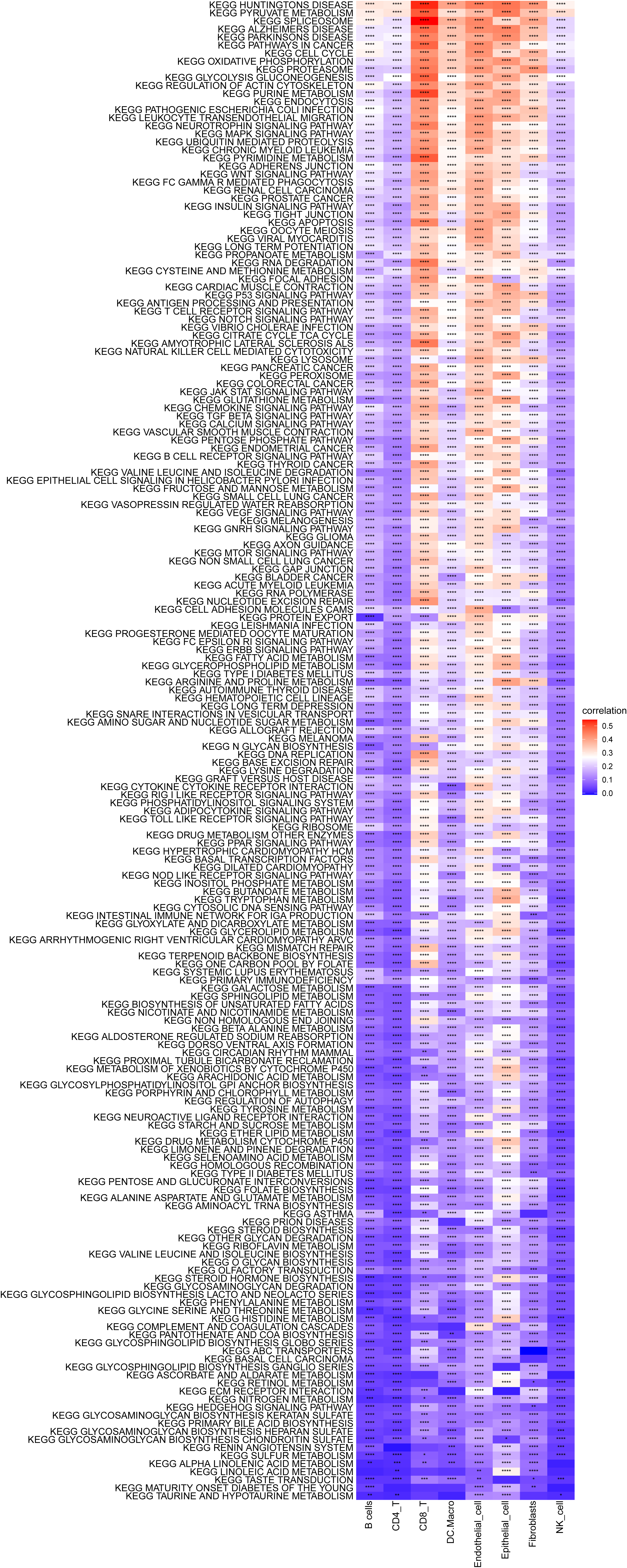

Supplement: Supplementary file 7 — Additional file 7: Figure S6. Heatmap of correlation between lactylation score and the KEGG pathways in each cell type. Each cell was scored based on the KEGG_MEDICUS subset of Canonical pathways (MsigDB database) and the correlation of the Lactylation score with the KEGG pathway score is shown. [file 12967_2024_5092_MOESM7_ESM.tif]

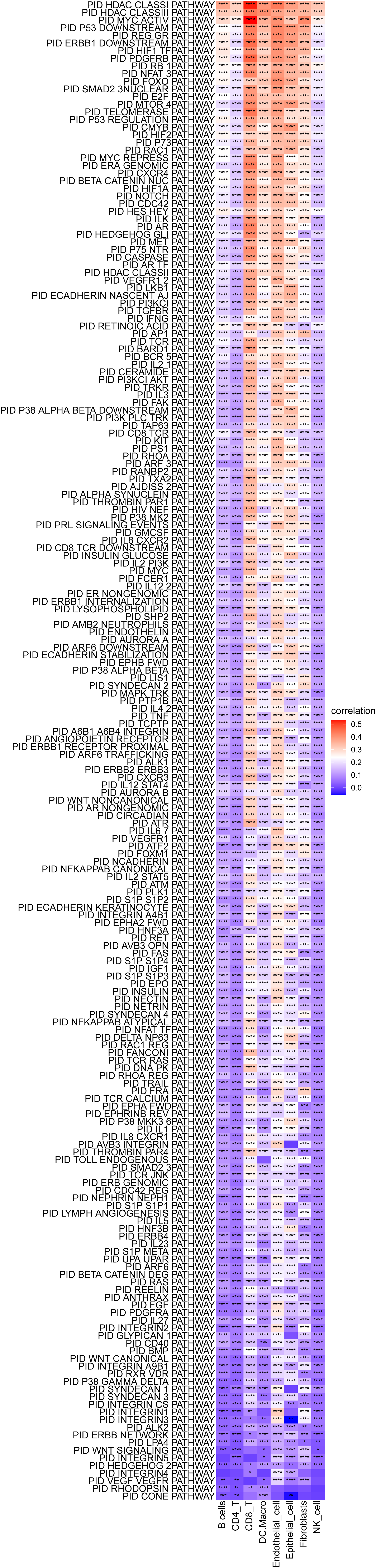

Supplement: Supplementary file 8 — Additional file 8: Figure S7. Heatmap of correlation between lactylation score and the PID pathways in each cell type. Each cell was scored based on the PID subset of Canonical pathways (MsigDB database) and the correlation of the Lactylation score with the PID pathway score is shown. [file 12967_2024_5092_MOESM8_ESM.tif]
